# Estimating productivity levels in primary medical services across clinical commissioning groups in England and the impact of the COVID-19 pandemic: a data envelopment analysis

**DOI:** 10.1186/s12913-023-10117-2

**Published:** 2023-11-02

**Authors:** Kate Williams, Stacey Croft, Mohammed A. Mohammed, Steven Wyatt

**Affiliations:** 1 Department of Mathematics, University of Birmingham, Birmingham, UK; 2 The Strategy Unit, NHS Midlands and Lancashire Commissioning Support Unit, Birmingham, West Midlands UK; 3 Faculty of Health Studies, University of Bradford, Bradford, UK; 4 Applied Research Collaboration (ARC) West Midlands, Institute of Applied Health Research, Murray Learning Centre, University of Birmingham, Birmingham, West Midlands UK

**Keywords:** Primary medical services, General practice, Productivity, Efficiency, COVID-19

## Abstract

**Objectives:**

To assess the relative productivity of primary medical services in England and the impact of the COVID-19 pandemic on productivity levels.

**Setting:**

Primary medical services for 59 million patients (98% of the population in England), in 101 clinical commissioning groups (CCGs), across two time periods: period 1, pre-pandemic, April to December 2019 and period 2, pandemic, April to December 2020.

**Methods:**

We use data envelopment analysis (DEA) to assess relative productivity with four input measures (the number of full-time equivalent general practitioners, nurses, other direct patient contact staff and administrators), and five output measures (face-to-face appointments, remote consultations, home visits, referrals to secondary care and prescriptions). Our units of analysis were CCGs. DEA assigns an efficiency score to a CCG, taking a value between 0 and 100%, by benchmarking it against the most productive CCGs. We use Tobit regression to examine the association between productivity and other factors.

**Results:**

The mean bias-corrected efficiency score of primary medical services in CCGs was 92.9% (interquartile range 92.0% to 95.7%) in period 1, falling to 90.6% (interquartile range 86.8% to 95.2%) in period 2. In period 1, CCGs with a higher proportion of registered patients aged over 65 years, higher levels of deprivation, lower levels of disease prevalence, higher nurse to GP ratios and higher GP to other direct patient contact staff ratios, achieved statistically significantly higher general practice efficiency scores (*p* < 0.05). In period 2, only the ratio of GP to other direct patient contact staff was associated with efficiency scores (*p* > 0.05).

**Conclusions:**

Our analysis indicates only modest geographic variation in productivity of primary medical services when measured at the level of clinical commissioning groups and a small reduction in productivity during the pandemic. Further work to establish relative productivity of individual GP practices is warranted once sufficient data on appointment rates by GP practice is available.

**Supplementary Information:**

The online version contains supplementary material available at 10.1186/s12913-023-10117-2.

## Introduction

Primary medical services in England are delivered by approximately 7000 general practices, each providing care to their registered patients [1]. They diagnose and treat patients’ acute healthcare needs, manage patients’ long-term conditions, prescribe medicines, deliver screening and immunisation programmes and refer patients to secondary care when their condition requires more specialised attention. Until July 2022, each practice was a member of one of 106 geographically defined NHS Clinical Commissioning Groups (CCGs), the statutory bodies tasked with commissioning secondary and other health services for a local population. CCGs have now been superseded by Integrated Care Boards.

Approximately 9% of government spending on health in England is allocated to primary care [2]. 20 to 30 million appointments are offered to patients each month, taking the form of face-to-face consultations, home visits, telephone, video and online interactions with general practitioners, nurses and other healthcare professionals [3]. Annual patient surveys indicate high levels of satisfaction, although patient’s frequently report difficulties contacting their practice and booking an appointment [4].

The COVID-19 pandemic had a significant impact on primary care. To limit the spread of the disease, practices were initially instructed by NHS England to triage patients by telephone, and to conduct consultations remotely where possible [5, 6]. Meanwhile, patients were advised to stay at home and limit social interactions [7]. The frequency of primary care appointments reduced substantially during the period from the end of March 2020 to September 2020, with increases in telephone appointments offsetting some of the reduction in face-to-face consultations [8]. Further disruptions came in the form of the COVID-19 vaccination programme. GP practices were one of the main routes by which COVID-19 vaccines were made available to the English population in 2021.

The pressures on GP services, and in particular the challenge in meeting rising demand from patients, have been widely reported [9–12]. These are not new, but have intensified since Government restrictions on social mixing were lifted. In 2022, 47% of patients reported difficulties making contact with their GP practice by phone, up from 30% in 2018 [4]. A wide range of policies and initiatives have attempted to address this issue, by increasing staffing levels, extending opening hours, developing and deploying online tools, federating practices, and improving productivity and efficiency [13–15].

Attempts to measure the productivity of General Practices in England have been hampered by a lack of data on one of the key output measures, patient consultations [16]. We use a new dataset, containing data on the rates of GP appointments, alongside existing data about other practice inputs and outputs, to assess the relative productivity of primary care across CCGs in England in 2019 and 2020, using data envelopment analysis (DEA). We sought to determine the extent of variation in productivity across CCGs and the impact of the pandemic on productivity levels.

## Methods

### Setting and population

Our analysis examines the relative productivity of primary medical services in England. Our units of analysis are CCGs. Each general practice is a member of one CCG. Our analysis is conducted at this level, rather than at the level of general practices, since data on counts of appointments are aggregated at CCG level before publication. We excluded 6 (4.7%) CCGs representing 2.0% of registered patients due to incompleteness of appointment data (see Supplementary file 1).

Our analysis was conducted over two 9-month periods: April 2019 to December 2019, prior to the COVID-19 outbreak in the UK, and April 2020 to December 2020, the 9-month period following the outbreak. Some of the published datasets used to construct input and output variables were aggregated at the level of calendar quarters, constraining the selection of time-periods for analysis. We chose a 9-month rather than 12-month period because GP practice activity was substantially affected by the COVID-19 vaccination programme after December 2020.

### Variables and data sources

Our data envelopment analysis used 4 input variables, the number of full-time equivalent (FTE) general practitioners (including partners, salaried GPs, trainees and locums), nurses, other clinical staff and administrative staff, and 5 output variables, the number of face-to-face and telephone appointments, home visits, secondary care referrals and prescriptions issued. Workforce, appointment and prescribing data was obtained from NHS Digital [17–19]. Data on referrals to secondary care were obtained from NHS England [20].

We regressed the resulting efficiency scores against 9 independent variables, selected to represent the size, and health needs of the registered populations, and the staffing skill-mix in each CCG and time period: (1) the number of registered patients, (2) the proportion of this population aged 65 years or more, (3) the mortality and (4) fertility rate, (5) the level of deprivation and (6) the prevalence of several long-term conditions, (7) the ratio of FTE GPs to nurses, (8) other direct patient contact staff and (9) administrative staff. Data on the number of registered patients and their age profile, the fertility and mortality rates were obtained from Public Health England [21]. Deprivation was measured using the English Indices of Deprivation 2019 obtained from the Ministries of Housing, Communities and Local Government [22]. Data on the reported prevalence of 20 conditions were obtained from NHS Digital: atrial fibrillation, asthma, cancer, coronary heart disease, chronic kidney disease, chronic obstructive pulmonary disease, dementia, depression, diabetes, epilepsy, heart failure, hypertension, learning disability, severe mental illness, obesity, osteoporosis, peripheral arterial disease, palliative care, stroke and transient ischaemic attack, and rheumatoid arthritis [23].

### Statistical methods

Data on staffing levels in GP practices are published on a quarterly basis. Over the 6 quarters of interest, a small proportion of practices failed to report the number of GPs (0.8%), nurses (2.6%), other direct patient contact staff (4.8%) and administrative staff (0.1%). These missing values were imputed by regressing the number of FTE staff by type against the registered population.

Data on the monthly count of appointments, published at CCG level, were adjusted to take account of the fact that not all practices reported data each month (3.6% missing), and a proportion (4.7%) of appointments were marked as having unknown appointment mode (face to face, telephone, video, or home visit) for each CCG. CCGs were excluded from the analysis if no information on the appointment mode was available, or if the appointment mode was unknown in more than 40% of appointments.

Data on staffing levels, prescriptions, and disease prevalence were sourced at the level of GP practices, and later aggregated to the level of CCGs using data on GP practice CCG membership from NHS Digital. Many CCGs underwent reconfigurations and mergers during the study period. All data were reframed into the latest CCG configuration using information on successor organisations from NHS Digital.

Data envelopment analysis (DEA) is a non-parametric, deterministic form of frontier analysis which can be used to estimate the relative efficiency of a set of decision-making units (DMUs), in our case CCGs [24]. The method can accommodate multiple inputs and outputs and does not require prior knowledge about the relationship between these variables. DEA assigns an efficiency score between 0 and 1 to each of the DMUs, with a score of 1 meaning that the DMU is fully efficient, i.e. “none of its inputs or outputs can be improved without worsening some of its other inputs or outputs".^25^ We use the output orientation of DEA, since we wished to estimate the additional outputs that could be delivered given the current input levels, and assumed variable returns to scale (VRS).

A window DEA technique was used to evaluate the DMUs over two time periods, by allowing the entities to be evaluated as different DMUs in each time period. Since data envelopment analysis is deterministic, it can be sensitive to measurement errors. A form of bootstrapping, described by Simar and Wilson, is used to find bias corrected efficiency scores [25]. The change in a DMU’s productivity over time is found using the Malmquist productivity index [26]. This is defined by distance functions which can be found using the calculated efficiency scores [27].

Tobit regression was then used to regress the calculated efficiency scores against factors which may impact on efficiency. Tobit regression was used since our dependent variable, the bias corrected efficiency score, is right-censored. Independent variables were scaled to aid interpretation of the model coefficients. Our models were stratified by time period.

We used k-medoids clustering to assign a CCG to one of three groups based on its disease prevalence rates [28]. These groups were labelled following a descriptive analysis of the results as (1) low disease prevalence, (2) high prevalence of strongly age-related conditions (e.g., chronic obstructive pulmonary disease, dementia, heart failure, osteoporosis) and (3) high prevalence of other conditions (e.g., obesity, severe mental illness, asthma, epilepsy)—see S1 table in the Supplementary file 1. The resulting assignment was used as a design (dummy) variable in our regression, along with the number of registered patients, proportion of patients aged over 65 years, deprivation, birth rate, death rate, FTE GP to nurse ratio, FTE GP to other clinical staff ratio and FTE GP to admin ratio.

All analyses were undertaken using R version 4.0.3 and the Benchmarking, Tidyverse, VGAM and Cluster packages [29–33].

## Results

### Description of primary medical services

In Table 1, we set out the characteristics of primary medical services across the 101 CCGs included in our study, in the two 9-month periods before and after the outbreak of COVID-19 in England. On average, CCGs delivered primary medical services to 580,000 patients each, in 67 practices, with 324 FTE GPs, 160 FTE nurses, 121 FTE other clinical staff and 652 FTE administrative staff. These figures increased marginally between period 1 and period 2, with the largest proportional increases seen in the numbers of FTE other clinical staff.

**Table 1:** Characteristics of primary medical services in 101 CCGs

	Period 1 mean (sd)	Period 2 mean (sd)	% change
Registered patients	582,213 (494,917)	586,736 (501,467)	+ 0.8%
GP practices	67 (60)	65 (57)	-3.4%
GPs FTE	324 (264)	329 (266)	+ 1.5%
Nurses FTE	160 (115)	162 (117)	+ 1.3%
Other clinical staff FTE	121 (97)	132 (107)	+ 9.1%
Administrative FTE	652 (494)	661 (499)	+ 1.5%
Face-to-face appointments	1,927,391 (1,508,782)	1,087,314 (854,352)	-43.6%
Telephone/video appointments	334,614 (305,959)	870,530 (744,245)	+ 160.2%
Home visits	22,939 (23,578)	12,354 (12,023)	-46.1%
Referrals	93,472 (82,295)	57,604 (47,776)	-38.4%
Prescriptions	826,169 (642,817)	795,374 (613,704)	-3.7%

Period 1 – Apr to Dec 2019, period 2 – Apr to Dec 2020, FTE – full time equivalent

In period 1, primary medical services in an average CCG delivered 1.93 million face-to-face appointments, 330 thousand telephone or video appointments, 23 thousand home visits, 93 thousand referrals to secondary care and 826 thousand prescriptions. Between period 1 and period 2, the mean number of face-to-face appointments per CCG reduced by 840 thousand (-43.6%), whilst home visits reduced by 11 thousand (-46.1%), and referrals by 36 thousand (-38.4%). Mean prescriptions per CCG reduced more modestly by 31 thousand (-3.7%) whilst telephone or video appointments increased by 536 thousand (+ 160.2%).

### Efficiency of primary medical services

**Fig. 1:**
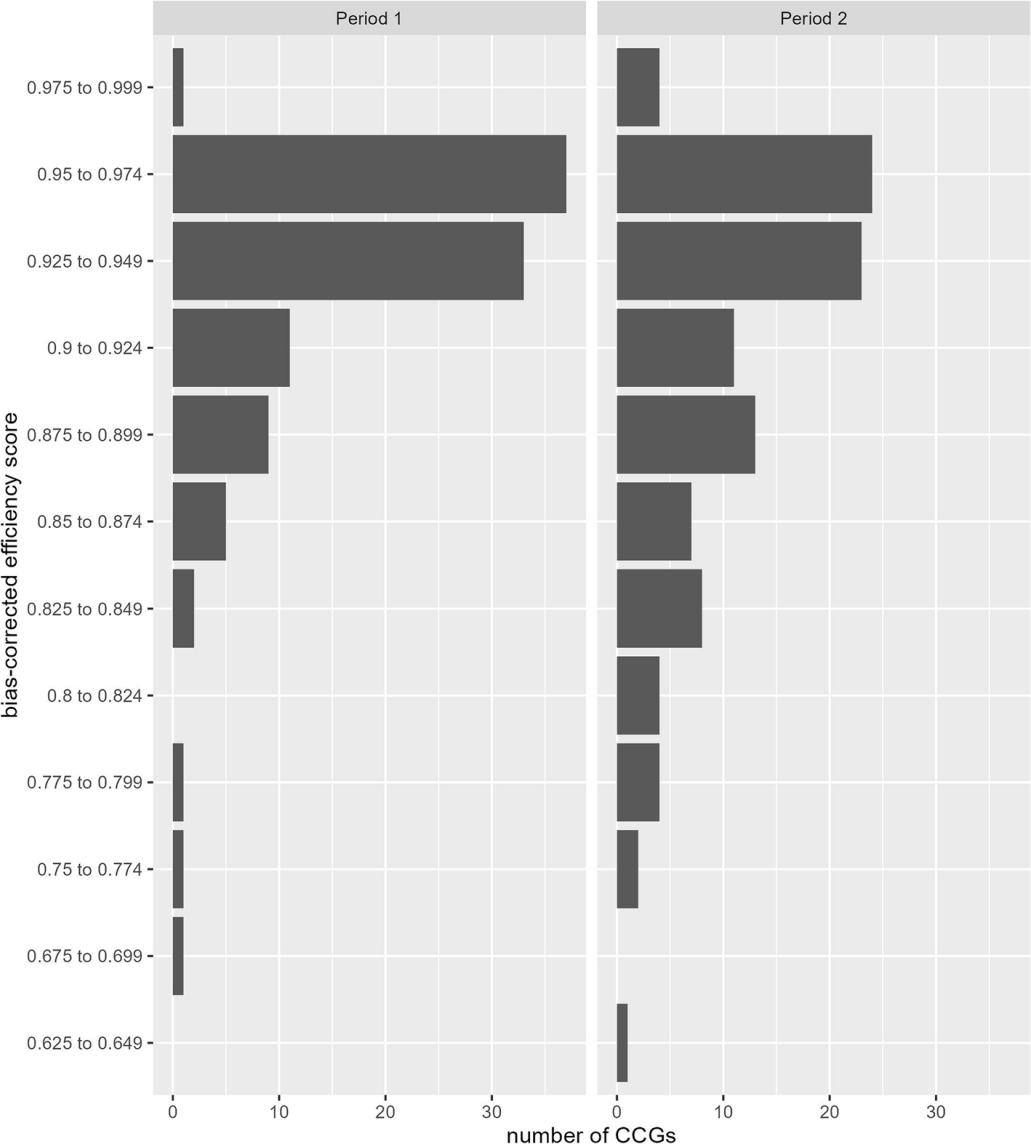
CCG frequency of bias-corrected efficiency scores

Table 2 and Fig. 1 shows the frequency of CCGs by bias-corrected efficiency scores in periods 1 and 2. The mean efficiency score of CCGs was 92.9% (interquartile range 92.0% to 95.7%) in period 1, falling to 90.6% (interquartile range 86.8% to 95.2%) in period 2.

**Table 2:** Frequency of primary medical services bias-corrected efficiency scores in 101 CCGs

Bias-corrected efficiency scores	Period 1 Number of CCGs	Period 2 Number of CCGs
0.625 to 0.649	0	1
0.675 to 0.699	1	0
0.750 to 0.774	1	2
0.775 to 0.799	1	4
0.800 to 0.824	0	4
0.825 to 0.849	2	8
0.850 to 0.874	5	7
0.875 to 0.899	9	13
0.900 to 0.924	11	11
0.925 to 0.949	33	23
0.950 to 0.974	37	24
0.975 to 0.999	1	4

Period 1 – Apr to Dec 2019, period 2 – Apr to Dec 2020

For inefficient CCGs in period 1, weighted efficient peers are drawn almost exclusively (98.5%) from period 1. The opposite is true for period 2, where 84.0% of weighted efficient peers are drawn from period 2. This suggests two largely distinct frontiers exist, before and during the pandemic.

In Table 3 we show the estimated additional outputs that CCGs might have delivered if they had operated in line with their most efficient peers and without increasing their staffing levels. During period 1, the CCGs might have delivered an additional 7.53 million (+ 3.9%) face-to-face appointments, 1.86 million (+ 5.5%) telephone and video appointments, 557 thousand (+ 24.1%) home visits, 705 thousand (+ 7.5%) referrals, and 6.74 million (+ 8.1%) prescriptions. In period 2 the efficiency opportunities were larger: + 10.7% for face-to-face appointments, + 6.2% for telephone and video appointments, + 35.1% for home visits, + 19.5% for referrals, and + 12.3% for prescriptions.

**Table 3:** Potential additional outputs

Output	Period1	Period2
Face-to-face appointments	7,526,995 (3.9%)	11,791,630 (10.7%)
Telephone/video appointments	1,866,292 (5.5%)	5,447,448 (6.2%)
Home visits	557,317 (24.1%)	438,146 (35.1%)
Referrals	705,159 (7.5%)	1,134,289 (19.5%)
Prescriptions	6,742,452 (8.1%)	9,920,685 (12.3%)

Period 1 – Apr to Dec 2019, period 2 – Apr to Dec 2020

### Changes in efficiency before and after the COVID-19 outbreak

**Fig. 2:**
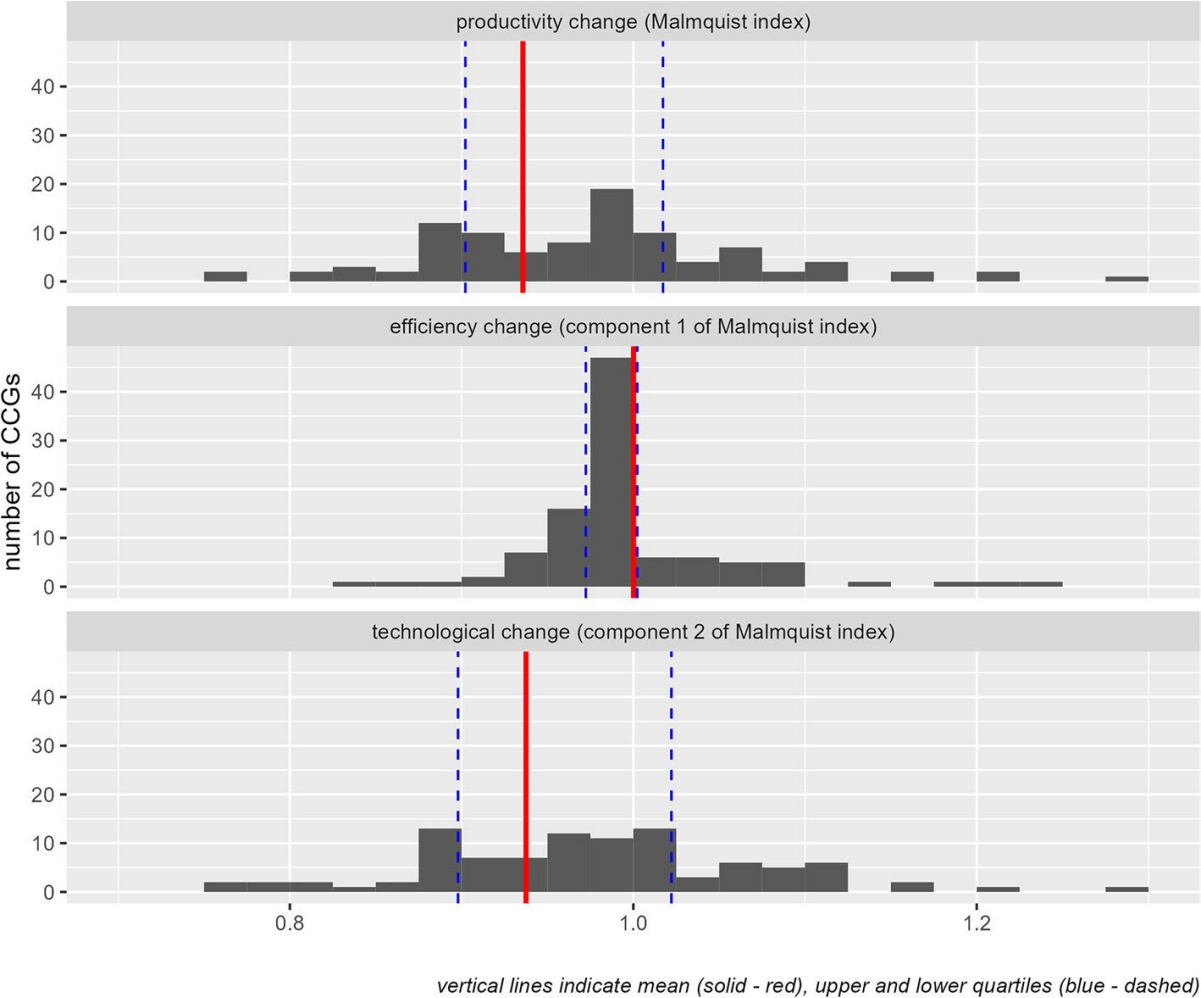
Change in productivity from period 1 to period 2 | 101 CCGs)

We compare the efficiency of each CCG before and after the pandemic using the Malmquist Index. This links a CCG’s observations over time to estimate the change in productivity between period 1 and period 2. Figure 2 illustrates the distribution of these CCG-level changes in productivity. The mean Malmquist index was 0.936 (i.e., a reduction in productivity of 6.4%) with inter-quartile range 0.902 to 1.017. The Malmquist index can be seen as the product of two factors: efficiency change and technological change. Efficiency change measures the change in each CCG’s efficiency score if there had been no change in the efficient frontier. Technological change measures the impact of changes in the technological frontier (operating models and associated technology) on a CCGs measured efficiency. Our analysis suggests the CCG mean efficiency change component of the Malmquist index was 1.000 (i.e., no change) with inter-quartile range from 0.971 to 1.002. The CCG mean technological change component was 0.938 (inter-quartile range 0.898 to 1.022).

### Factors associated with the efficiency of primary medical services

In our pre-pandemic period, we found that CCGs with a higher proportion of registered patients aged over 65, higher levels of deprivation, lower levels of disease prevalence, higher nurse to GP ratios and higher GP to other direct patient contact staff ratios, achieved statistically significantly higher general practice efficiency scores (*p* < 0.05, see Table 4). In the pandemic period, only the last of these significant associations with efficiency (i.e., with the ratio of GP to other direct patient contact staff) was preserved. Having controlled for these variables we found no association between efficiency and birth or death rates, practice list size, or the ratio of GPs to administrative staff.

**Table 4:** Tobit regression results

	Period 1			Period 2		
Model term	Estimate	95% CI	*P* > |z|	Estimate	95% CI	*P* > |z|
Intercept	0.682	0.468, 0.896	0.000	0.704	0.396, 1.013	0.000
1k registered patients	0.001	-0.001, 0.003	0.529	0.000	-0.003, 0.003	0.869
% patients aged 65+ yrs	0.012	0.002, 0.021	0.017	0.008	-0.004, 0.020	0.205
Deprivation (IMD2019)	0.002	0.000, 0.004	0.042	0.003	0.000, 0.006	0.073
Births per 1k patients	0.008	-0.001, 0.018	0.085	0.008	-0.007, 0.022	0.292
Deaths per 1k patients	-0.016	-0.032, 0.001	0.064	-0.013	-0.030, 0.004	0.146
Disease prevalence						
High prev. a-r (ref)	0.000	-	-	0.000	-	-
High prev. other	0.040	0.005, 0.075	0.026	0.043	-0.002, 0.088	0.062
Low prev.	-0.018	-0.045, 0.010	0.219	0.003	-0.037, 0.044	0.876
GP:Nurse ratio	-0.034	-0.064, -0.004	0.029	-0.016	-0.055, 0.023	0.427
GP:OtherDPC ratio	0.019	0.009, 0.030	0.000	0.022	0.004, 0.040	0.018
GP:Admin ratio	-0.002	-0.213, 0.209	0.986	-0.035	-0.304, 0.234	0.799

Period 1 – Apr to Dec 2019, period 2 – Apr to Dec 2020, a-r – age-related conditions, DPC – direct patient contact staff

Estimates are adjusted for the number of registered patients, proportion of patients aged over 65 years, deprivation, birth rate, death rate, disease prevalence cluster, FTE GP to nurse ratio, FTE GP to other clinical staff ratio and FTE GP to admin ratio

## Discussion

### Summary of main findings

Our analysis indicates a modest level of variation in the productivity of primary medical services across CCGs. If these productivity differences were eliminated, then this analysis suggests that the number of face-to-face appointments in the period from April to December 2019 could have been increased by 3.9% without changing staffing levels or skill mix. Larger increases could have been delivered in the other outputs considered, remote consultations, home visits, referrals, and prescriptions, and during the period from April to December 2020.

The COVID-19 pandemic and associated guidance for patients and practices, generated a significant change in the pattern of GP practice provision and therefore in the shape of the production frontier. Increases in remote consultations and reductions in face-to-face consultations were ubiquitous. We found that these changes were associated with modest reductions in average productivity levels, although some CCGs were able to buck this trend and secure productivity increases. A recent mixed methods study of the rapid switch to remote working in primary care in the early period of the pandemic, concluded that the change was widely supported, and enabled practices to manage patient’s needs whilst minimising the spread of COVID-19 [34]. Although data on sickness absence amongst general practice staff is limited, it seems likely that these were elevated during the pandemic. It is in this context that our findings about modest productivity reductions during the pandemic, must be viewed.

CCGs with a higher proportion of registered patients aged over 65 and higher levels of deprivation tended to achieve higher levels of productivity in the pre-pandemic period. These CCGs may have been under greater pressure to increase outputs in response to patient demand, and it is possible that this has given greater impetus to improve productivity. Further work would be required to confirm this theory. We also found that CCGs with higher nurse to GP ratios were more productive. This may be explained by the fact that the cost of employing a nurse is lower than employing a GP. All other things equal, CCGs with higher nurse to GPs ratios would therefore have more staff and more time to deliver patient appointments. Not all consultations are of equal value however, and our analysis did not distinguish between nurse and GP consultations.

The positive association between productivity and the ratio of GPs to other direct patient contact staff, is more difficult to explain. Taken at face value, this finding suggests that practices should not increase the number of other direct patient contact staff if they wish to improve their productivity. If, however, this staff group conducts activities that are not well represented by the outputs considered in this study, then this would understate their impact on productivity. Furthermore, the heterogeneity of this staff group limits the interpretability and utility of this result.

### Relationship with existing literature

Two systematic reviews of primary care efficiency measurement highlight the challenge of measuring outcomes in primary care, and point out that most studies rely either on outputs, as with this study, or quality measures [35, 36]. Although focused on performance rather than productivity of primary care, an English 2001 study found that results were sensitive to the specification of the production process [37]. A study of the efficiency of primary care services across 20 regions in Italy, reported mean efficiency scores of 95%, and a negative association between efficiency and service expenditure [38]. A series of publications produced by the University of York, provide longitudinal estimates of NHS productivity. In the most recent publication, the authors estimate that cost-weighted productivity of primary medical services reduced by 5.0% between 2019/20 and 2020/21 [39]. Although derived using a different method (Laspeyres cost weighted measurement), with different assumptions, and covering slightly different time periods, this figure is broadly comparable with our estimate of productivity changes before and during the COVID-19 pandemic. A recent paper finds that continuity of care leads to productivity gains by increasing the interval between patient consultations, thereby reducing demand [40].

### Limitations of the study

NHS Digital, the source of our appointments data, highlights several issues relating to data reliability and quality. They point out that the data does not show the totality of GP activity or workload, rather only that which is captured on GP practice information systems. Whilst coverage of the input and output data was high, there were some gaps. These were filled with imputation methods that may introduce bias. Our disease prevalence data was obtained from NHS Digital’s publication of the Quality and Outcomes Framework (QOF). NHS Digital suggests that the recording of QOF activity may have been affected by the pandemic and whilst participation in QOF is high, involvement is voluntary [41].

Our analysis was carried out at the level of CCGs rather than GP practices, because our appointments data were grouped at this level before publication. Whilst this may reduce risks associated with poor data quality in individual practices, it is likely to obscure the true level of productivity variation in primary medical services.

For DEA to be effective, the number of inputs and outputs must be small relative to the number of service units. One of the consequences therefore of analysing productivity using DEA across 101 CCGs (rather than c. 7000 practices), is to constrain the number of inputs and outputs that could be considered. This is a significant limitation. Any conclusions drawn from the analysis must acknowledge the limited and undifferentiated nature of the service inputs and outputs that were considered. The analysis does however illustrate that the approach is feasible and has potential and when sufficient GP practice level data on appointments becomes available, revisiting this analysis, expanding the types of input and output to create a more complete and rounded assessment of GP practice productivity, would be warranted.

Our study focuses on the quantity rather than on the quality or outcomes of care. The quantity of outputs almost certainly lies on the causal path between investment in primary care and its outcomes. Understanding and quantifying changes and variation in productivity is therefore of value, even if it does not solely determine patient outcomes. Moreover, it is the shortage of outputs, and in particular, appointments, that is exercising services and policy makers at present.

The method used in the paper, DEA, measures productivity opportunities by examining variation in input–output ratios between units. The frame of reference is necessarily limited by extant service models. Opportunities to improve productivity through the introduction of novel technologies and service models are therefore out of view.

### Policy and practice implications

Given the limitations of the study, we suggest it should primarily be seen as a proof of concept for DEA based assessments of the productivity of primary medical services in England. We note that since November 2022, NHS Digital has published appointments data aggregated at the level of GP practices. More robust and detailed analysis of GP practice productivity will be feasible once enough of this data has accumulated. A DEA analysis based on data for several thousand practices would allow for greater granularity of inputs and outputs. This data would also support complex quality adjustments, such as those developed by Arabadzhyan.^40^

DEA defines a production frontier made up of the most productive services and against which all other services are compared. The distance between DMUs and the production frontier represents potential productivity gains. This analysis suggests that this productivity potential is present but modest in size. Realising this potential will require skilful engagement with practices and appropriate quality improvement methods. Furthermore, the methodology has little to say about the sustainability of services on the frontier. Given the reported pressures on GP services, caution should be exercised before setting expectations of productivity improvements for services close to this production frontier.

The analysis found that there were reductions in the productivity of primary medical services between 2019 and 2020, corroborating the findings of others. These productivity losses lead to reduced outputs and manifest as patients expressing concern about service access. The most recent national policy for primary care seeks to address this access challenge through several strategies including improvements in productivity [42]. Given the perennial nature of this challenge, policy makers should evaluate the adoption and impact of the strategy so that lessons can be learnt. In particular, the evaluations should track changes in productivity and productivity variation.

## Conclusion

Our analysis indicates only modest variation in productivity of primary medical services when measured at the level of clinical commissioning groups and a marginal reduction in productivity during the pandemic. Further work to establish relative productivity of individual GP practices is warranted once sufficient data on appointment rates by GP practices becomes available.

### Supplementary Information

**Additional file 1.**

## Data Availability

The raw data used is this study are available from the following open access websites.

**Table:** 

workforce	https://digital.nhs.uk/data-and-information/publications/statistical/general-and-personal-medical-services
appointments	https://digital.nhs.uk/data-and-information/publications/statistical/appointments-in-general-practice
prescriptions	https://digital.nhs.uk/data-and-information/areas-of-interest/prescribing/practice-level-prescribing-in-england-a-summary
referrals	https://www.england.nhs.uk/statistics/statistical-work-areas/outpatient-referrals/mrr-data/
population, births & deaths	https://fingertips.phe.org.uk
deprivation	https://www.gov.uk/government/statistics/english-indices-of-deprivation-2019
prevalence	https://digital.nhs.uk/data-and-information/publications/statistical/quality-and-outcomes-framework-achievement-prevalence-and-exceptions-data Tidied data files are available from the corresponding author on reasonable request.
