# Supplementary material for: Estimating productivity levels in primary medical services across clinical commissioning groups in England and the impact of the COVID-19 pandemic: a data envelopment analysis
Source: BMC Health Serv Res. 2023 Nov 2;23:1194. doi: 10.1186/s12913-023-10117-2 (PMC10621225; doi:10.1186/s12913-023-10117-2)
Supplement: Supplementary file 1 — Additional file 1. [file 12913_2023_10117_MOESM1_ESM.docx]

## Supplementary file 1

### CCG exclusions

Five CCGs were excluded from our analysis. One CCG was excluded because no appointment mode data (i.e., face to face, telephone/video or home visit) was available. A second CCG was excluded because the appointment mode data was not complete in more than 40% of cases. Two further CCGs were excluded because no home visiting data was available in either the pre-pandemic or pandemic period, or both. A fifth CCG was excluded because its home visiting rate per head of registered population was implausibly low (i.e., < 0.1% of the mean CCG rate).

### Descriptive analysis of CCG disease prevalence clusters

S1 Table: Mean CCG prevalence by disease and CCG disease prevalence clusters

| Condition group | Disease register | CCG clusters | | |
| --- | --- | --- | --- | --- |
|  |  | (1)  Low prevalence | (2)  High prevalence  age-related conditions | (3)  High prevalence other conditions |
| strongly age-related conditions | atrial fibrillation | 1.7% | 2.6% | 2.1% |
|  | cancer | 2.8% | 3.9% | 3.1% |
|  | chronic kidney disease | 2.6% | 4.0% | 3.4% |
|  | chronic obstructive pulmonary disease | 1.6% | 2.3% | 2.6% |
|  | coronary heart disease | 2.5% | 3.8% | 3.6% |
|  | dementia | 0.6% | 0.9% | 0.8% |
|  | diabetes | 5.3% | 6.0% | 6.3% |
|  | heart failure | 0.7% | 1.1% | 1.0% |
|  | hypertension | 12.7% | 16.1% | 15.0% |
|  | osteoporosis | 0.2% | 0.4% | 0.3% |
|  | palliative care | 0.4% | 0.6% | 0.5% |
|  | peripheral arterial disease | 0.5% | 0.7% | 0.8% |
|  | rheumatoid arthritis | 0.6% | 0.7% | 0.7% |
|  | stroke & transient ischaemic attack | 1.5% | 2.2% | 2.0% |
| other conditions | asthma | 5.7% | 7.0% | 6.8% |
|  | depression | 8.7% | 10.8% | 11.3% |
|  | epilepsy | 0.6% | 0.7% | 0.8% |
|  | learning disability | 0.5% | 0.5% | 0.6% |
|  | obesity | 5.8% | 7.9% | 8.2% |
|  | severe mental illness | 0.9% | 0.9% | 1.0% |
